# Supplementary material for: Lower airway microbiota compositions and diversity among ventilator-associated pneumonia patients across COVID-19 epidemic phases: a retrospective study
Source: Microbiol Spectr. 2025 Sep 19;13(11):e00076-25. doi: 10.1128/spectrum.00076-25 (PMC12584767; doi:10.1128/spectrum.00076-25)
Supplement: Supplemental figures — Fig. S1 and S2. [file spectrum.00076-25-s0001.docx]

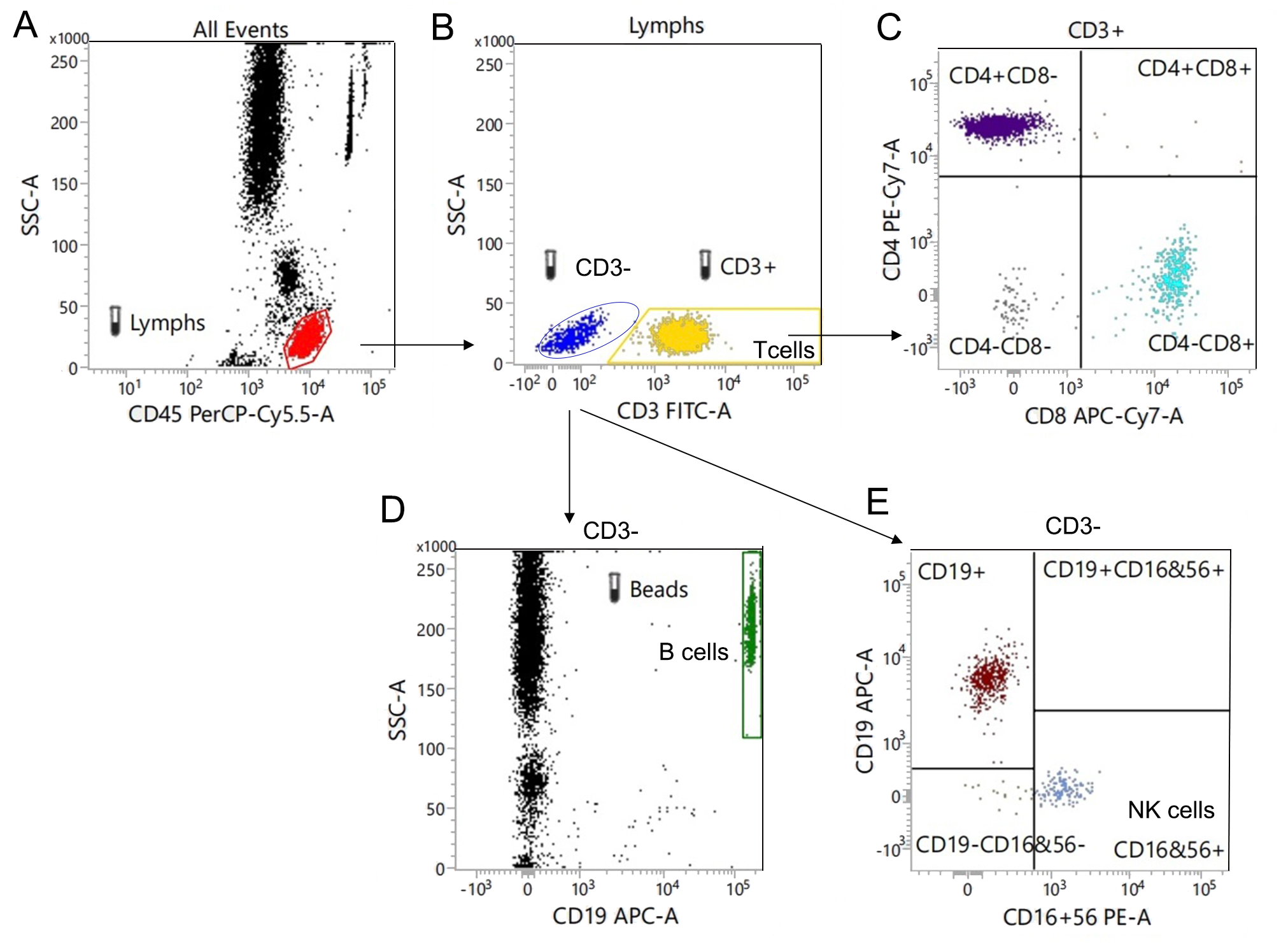


Supplementary Fig 1. Gating strategy for lymphocyte subpopulations. **A.** Identification of CD45⁺ lymphocytes from total events. B. CD3⁺ T cells and CD3⁻ non-T cells gated within CD45⁺ lymphocytes. **C.** Subsetting of CD3⁺ T cells into CD4⁺CD8⁻ T helper (Th) cells and CD4⁻CD8⁺ cytotoxic T (Tc) cells using CD4 vs. CD8. D. Gating of CD19⁺ B cells from CD3⁻ non-T cells. E. Discrimination of B cells (CD19⁺) and NK cells (CD19⁻CD16⁺CD56⁺) via CD19 vs. CD16/CD56.


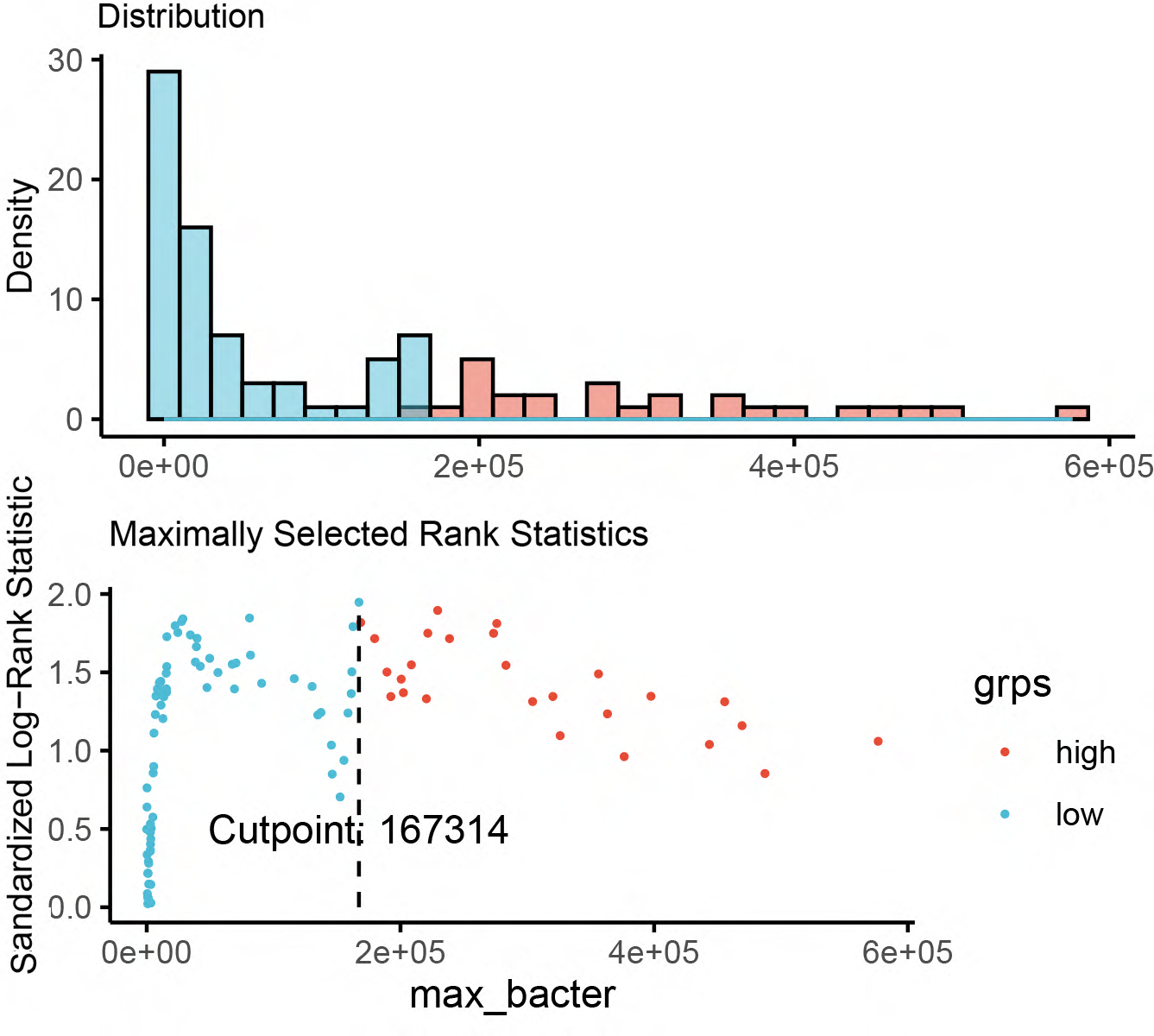
Supplementary Fig 2. Determination of optimal cutpoint for max_bacter based on maximally selected rank statistics method. The upper panel shows the distribution of (max_bacter). Each bar is colored according to the grouping determined by the optimal cutpoint. The lower panel illustrates the standardized log-rank statistics from the maximally selected rank statistics method, identifying the cutpoint that best separates the survival outcomes. The optimal cutpoint was found at max_bacter = 167,314, dividing patients into “low” (blue) and “high” (red) groups for subsequent analysis.
