# Supplementary material for: Lower airway microbiota compositions and diversity among ventilator-associated pneumonia patients across COVID-19 epidemic phases: a retrospective study
Source: Microbiol Spectr. 2025 Sep 19;13(11):e00076-25. doi: 10.1128/spectrum.00076-25 (PMC12584767; doi:10.1128/spectrum.00076-25)
Supplement: Supplemental tables — Tables S1 and S2. [file spectrum.00076-25-s0002.docx]

Supplementary Table 1. Demographic and clinical characteristics of patients.

| Total (n=175) | Pre（n=58） | |  | During（n=58） | |  | Post（n=59） | |  | Total |
| --- | --- | --- | --- | --- | --- | --- | --- | --- | --- | --- |
|  | Sur（n=30） | Non-sur（n=28） | **P value** | Sur（n=23） | Non-sur（n=35） | **P value** | Sur（n=42） | Non-sur（n=17） | **P value** | **P value** |
| Age, years | 58(42,66) | 69(61,74) | <0.01 | 67(62,75) | 78(70,81) | <0.05 | 61(50,70) | 63(55,73) | 0.308 | <0.001^*§^ |
| Gender (male), n (%) | 22(73.3) | 19(67.9) | 0.866 | 19(82.6) | 24(68.6) | 0.375 | 29(69.0) | 14(82.4) | 0.353 | 0.915 |
| BMI (kg/m^2^) | 22.5(18.9,24.6) | 22.1(21.1,24.3) | 0.844 | 23.0(20.4,27.1) | 23.4(7.9,25.9) | 0.363 | 22.0(19.9,26.7) | 23.7(22.2,25.7） | 0.454 | 0.240 |
| APACHE II score | 14(10,21) | 22(17,26) | <0.001 | 9(8,15) | 20(14,25) | <0.001 | 15(11,19) | 18(12,23) | 0.079 | 0.225 |
| SOFA score | 4(2,6) | 10(7,12) | <0.001 | 3(2,4) | 7(5,10) | <0.001 | 6(3,7) | 8(6,10) | <0.001 | 0.710 |
| Charlton score | 2(1,3) | 3(1,3) | 0.351 | 3(2,4) | 3(2,4) | 0.458 | 3(2,4) | 3(2,5) | 0.394 | <0.01^*¶^ |
| LOS in hospital (day) | 26(19,46) | 24(8,53) | 0.681 | 30(20,44) | 21(16,31) | 0.505 | 27(20,47) | 24(20,30) | 0.102 | 0.515 |
| LOS in ICU (day) | 27(19,47) | 24(8,53 | 0.805 | 28(14,44) | 19(12,26) | 0.537 | 16(10,26) | 13(9,23) | 0.168 | 0.406 |
| ***Therapy, n (%)*** |  |  |  |  |  |  |  |  |  |  |
| Immunosuppressant or glucocorticoids therapy | 4(13.3) | 4(14.3) | 1.000 | 8(34.8) | 7(20.0) | 0.342 | 14(33.3) | 6(35.3) | 1.000 | <0.05^¶^ |
| CRRT | 4(13.3) | 10(35.7) | 0.092 | 3(13.0) | 12(34.3) | 0.133 | 5(11.9) | 8(47.1) | <0.01 | 0.889 |
| ***Initial diagnosis, n (%)*** |  |  |  |  |  |  |  |  |  |  |
| Pulmonary disease | 5(16.7) | 15(53.6) | <0.01 | 15(65.2) | 12(34.3) | <0.01 | 12(28.6) | 4(23.5) | 0.712 | <0.05^¶^ |
| Cardiovascular disease | 3(10.0) | 5(17.9) | 0.717 | 1(4.3) | 6(17.1) | 0.109 | 18(42.9) | 8(47.1) | 0.487 | <0.05^¶§^ |
| Kidney disease | 1(3.3) | 1(3.6) | 1.000 | 0 | 0 | 1.000 | 1(2.4) | 0 | 1.000 | 0.317 |
| Trauma or fracture | 3(10.0) | 3(10.7) | 1.000 | 0 | 3(8.6) | 0.261 | 1(2.4) | 0 | 1.000 | 0.054^§^ |
| cerebrovascular or nervous system disease | 4(13.3) | 1(3.6) | 0.192 | 2(8.7) | 5(14.3) | 0.450 | 2(4.8) | 0 | 0.515 | <0.05^§^ |
| Digestive system disease | 6(20.0) | 2(7.1) | 0.127 | 2(8.7) | 3(8.6) | 1.000 | 0 | 1(5.9) | 0.333 | <0.05^¶^ |
| Tumor | 4(13.3) | 0 | 0.055 | 2(8.7) | 5(14.3) | 0.450 | 6(14.3) | 3(17.6) | 0.730 | <0.05^§^ |
| SLE | 0 | 0 | / | 0 | 1(2.9) | 1.000 | 0 | 1(5.9) | 0.333 | / |
| others | 4(13.3) | 1(3.6) | 0.192 | 1(4.3) | 0 | 0.429 | 2(4.8) | 0 | 0.515 | 0.191 |
| **Immune response** |  |  |  |  |  |  |  |  |  |  |
| TNF (pg/mL) | 13.8[9.0,23.0] | 34.1[18.6,47.2] | <0.001 | 12.8[11.3,20.9] | 16.5[11.0,26.9] | 0.386 | 11.6[5.9,19.0] | 9.7[7.0,21.2] | 0.893 | <0.001^¶§^ |
| IL-1β (pg/mL) | 6.5[5.0,12.2} | 15.9[7.2,28.6] | <0.05 | 5.0[5.0,7.1] | 10.6[5.0,18.2] | <0.05 | 5.0[5.0,8.7] | 5.9[5.0,10.5] | 0.468 | <0.01^¶^ |
| IL-2R (U/mL) | 940.0[522.0,1604.0] | 1872.0[1129.0,4669.0] | <0.01 | 703.0[474.0,860.0] | 1207.0[717.0,2062.0] | <0.01 | 705.0[341.0,1448.0] | 671.0[472.0,1485.0] | 0.645 | <0.01^¶^ |
| IL-6 (pg/mL) | 23.2[8.9,59.9] | 132.0[58.0,824.0] | <0.001 | 25.1[6.2,72.4] | 126.0[35.2,336.0] | <0.01 | 25.6[11.0,69.6] | 106.0[9.6,194.0] | 0.071 | 0.061 |
| IL-8 (pg/mL) | 51.0[21.8,79.8] | 118.0[57.5,516.0] | <0.01 | 59.0[20.5,86.0] | 93.0[56.5,206.0] | <0.05 | 27.5[10.8,77.8] | 38.0[9.0,86.0] | 0.461 | <0.01^¶§^ |
| IL-10 (pg/mL) | 7.9[5.0,15.4] | 21.0[5.4,44.7] | 0.027855 | 5.0[5.0,8.9] | 3.9[5.0,42.2] | <0.01 | 5.8[5.0,10.3] | 6.0[5.0,13.8] | 0.839 | <0.05^¶^ |
| WBC (*10^9/L) | 9.6[7.2,12.2] | 13.5[5.7,18.0] | 0.133 | 9.7[6.2,13.6] | 11.7[7.8,15.8] | 0.418 | 10.8[7.4,18.1] | 9.8[5.8,14.6] | 0.288 | 0.978 |
| NE (*10^9/L) | 7.8[5.8,10.4] | 11.4[3.8,16.7] | 0.154 | 7.8[5.7,14.2] | 10.6[5.8,14.4] | 0.628 | 8.9[4.9,14.3] | 8.5[5.0,13.1] | 0.407 | 0.749 |
| LYM (*10^9/L) | 0.8[0.5,1.2] | 0.7[0.5,1.2] | 0.403 | 0.8[0.5,1.0] | 0.6[0.2,0.8] | 0.051 | 0.7[0.4,1.5] | 0.6[0.4,0.7] | 0.081 | 0.137 |
| PLT (*10^9/L) | 182.0[112.0,274.0] | 68.5[26.8,156.0] | <0.01 | 204.0[138.0,239.0] | 111.0[69.0,177.0] | <0.05 | 122.0[88.0,238.0] | 106.0[84.0,185.0] | 0.436 | 0.787 |
| CD3^-^CD19^+^ B cells (%) | 0[0,11.3] | 7.4[0,20.6] | 0.063 | 0[0,9.6] | 13.2[0,24.3] | <0.05 | 5.6[0,26.0] | 11.0[4.4,25.4] | 0.362 | 0.331 |
| CD3^+^ T cells (%) | 0[0,64.9] | 55.0[0,61.1] | 0.314 | 0[0,64.8] | 58.4[0,72.0] | 0.178 | 39.0[0,65.1] | 57.5[41.5,68.2] | 0.125 | 0.603 |
| CD3^+^CD4^+^ T cells (%) | 0[0,30.4] | 22.2[0,32.9] | 0.334 | 0[0,28.5] | 27.3[0,40.0] | 0.109 | 19.8[0,38.6] | 33.6[28.8,45.8] | 0.058 | 0.213 |
| CD3^+^CD8^+^ T cells (%) | 0[0,17.4] | 17.0[0,23.8] | 0.087 | 0[0,26.4] | 19.0[0,27.6] | 0.127 | 12.8[0,21.2] | 12.0[8.1,24.3] | 0.277 | 0.644 |
| CD3^-^CD16^+^CD56^+^ NK cells (%) | 0[0,7.0] | 7.0[0,17.7] | <0.05 | 0[0,11.5] | 8.0[0,12.3] | 0.160 | 3.4[0,14.0] | 11.2[8.1,18.9] | <0.05 | 0.496 |
| D-Dimer (mg/L) | 4.1[2.3,8.0] | 5.8[3.2,8.8] | 0.388 | 2.0[0.8,4.7] | 4.3[2.2,6.7] | 0.100 | 4.7[2.7,6.4] | 4.3[2.9,8.1] | 0.874 | 0.098 |
| CRP (mg/L) | 56.8[38.9,135.0] | 113.0[52.6,183.0] | 0.246 | 62.2[18.4,150.0] | 175.0[93.4,220.0] | <0.01 | 54.0[0,177.0] | 167.0[90.0,244.0] | <0.05 | 0.283 |
| PCT (ng/mL) | 0.7[0.2,3.7] | 2.6[0.7,13.7] | <0.05 | 0.3[0.1,0.8] | 2.0[0.4,5.7] | <0.001 | 1.5[0.5,7.6] | 3.8[1.2,14.9] | 0.195 | <0.001^*§^ |
| Ferritin (ng/ml) | 0[0,507.0] | 1062.0[0,2000.0] | <0.01 | 721.0[0,1757.0] | 808.0[0,1230.0] | 0.801 | 0[0,0] | 0[0,476.0] | 0.347 | <0.001^¶§^ |
| P/F (mmHg) | 355.0[252.0,394.0] | 252.0[147.0,380.0] | 0.062 | 303.0[168.0,370.0] | 177.0[133.0,230.0] | <0.05 | 290.0[202.0,355.0] | 229.0[120.0,351.0] | 0.203 | <0.01^*§^ |

Data are presented as median (IQR) or n/N (%). For continuous variables, a one-way analysis of variance (ANOVA) was performed for comparisons among the three groups, with Bonferroni correction applied. If the assumption of homogeneity of variance was not met, Welch’s ANOVA was used instead. For categorical variables, the chi-square test was used for group comparisons, with Bonferroni correction applied. If the chi-square test assumptions were not met, Fisher’s exact test was employed. BMI, body mass index; APACHE II, acute physiology and chronic health evaluation; SOFA, sequential organ failure assessment; ICU, intensive care unit; LOS, length of stay; CRRT, continuous renal replacement therapy; TNF-α, tumor necrosis factor-α; IL-1β, interleukin-1β; IL-2R, interleukin-2 receptor; IL-6, interleukin-6; IL-8, interleukin-8; IL-10, interleukin-10; WBC, white blood cell count; NE, neutrophil count; LYM, lymphocyte count; PLT, platelet count; CD19, cluster of differentiation 19; CD3, cluster of differentiation 3; CD4, cluster of differentiation 4; CD8, cluster of differentiation 8; CD56, cluster of differentiation 56; D-Dimer, d-dimer; CRP, c-reactive protein; PCT, procalcitonin; Ferritin, ferritin; P/F, ratio of arterial oxygen partial pressure to fraction of inspired oxygen; ^*^Pre and During show differences; ^¶^ Pre and Post show differences; ^§^ During and Post show differences.

Supplementary Table 2. Comparison of antimicrobial drug usage in patients across the epidemic phases

| **Total (n=175)** | **Pre (n = 58)** | **During (n = 58)** | **Post (n = 59)** | **P** |
| --- | --- | --- | --- | --- |
| Second generation cephalosporins, n (%) | 1 (1.72%) | 1 (1.72%) | 1 (1.69%) | 1.000 |
| Third generation cephalosporins, n (%) | 12 (20.69%) | 16 (27.6%) | 12 (20.34%) | 0.643 |
| Penicillins, n (%) | 5 (8.62%) | 5 (8.62%) | 3 (5.08%) | 0.767 |
| Quinolones, n (%) | 1 (1.72%) | 3 (5.17%) | 5 (8.47%) | 0.302 |
| Carbapenems, n (%) | 33 (56.9%) | 31 (53.4%) | 33 (55.93%) | 0.781 |
| Macrolides, n (%) | 1 (1.72%) | 0 (0.00%) | 0 (0.00%) | 0.322 |
| Glycopeptides, n (%) | 10 (17.2%) | 8 (13.8%) | 18 (30.51%) | 0.061 |
| Polymyxins, n (%) | 2 (3.45=%) | 7 (12.1%) | 3 (5.08%) | 0.243 |
| Oxazolidinones, n (%) | 2 (3.45%) | 2 (3.45%) | 4 (6.78%) | 0.732 |
| Glycylcyclines, n (%) | 4 (6.9%) | 3 (5.17%) | 2 (3.39%) | 0.638 |
| Tetracyclines, n (%) | 0 (0.00%) | 0 (0.00%) | 1 (1.69%) | 1.000 |
| Cyclic Lipopeptides, n (%) | 1 (1.72%) | 1 (1.72%) | 2 (3.39%) | 1.000 |
| Nitroimidazoles, n (%) | 0 (0.00%) | 0 (0.00%) | 3 (5.08%) | 0.107 |
| Sulfonamides, n (%) | 0 (0.00%) | 1 (1.72%) | 1 (1.69%) | 1.000 |
| Antifungal Drugs, n (%) | 5 (8.62%) | 7 (12.1%) | 9 (15.25%) | 0.581 |
| Antiviral Drugs, n (%) | 1 (1.72%) | 2 (3.45%) | 2 (3.39%) | 1.000 |
| Combination, n (%) | 20 (34.48%) | 22 (37.9%) | 33 (55.93%) | 0.048 |

Data are presented as frequencies and percentages, n (%). For categorical variables, the chi-square test was used for comparisons among the three groups. If the assumptions of the chi-square test were not met, Fisher’s exact test was employed. For variables with a significant overall test result, Bonferroni correction was applied for post-hoc pairwise comparisons.
